# Popular Journalism

**Published:** 1891-12-26

**Authors:** 


### Popular Journalism.

We have long felt that a few newspapers hardly appreciate the damage which they do by inserting paragraphs with catching headings whenever any one makes a complaint whether ill—or well—founded against a hospital. In a leading article which appeared in the *Pall Mall Gazette* of the 18th inst., the following statements are made:—"Mr. H. C. Burdett, who conducts a paper called The Hospital, calls our attention to a falling-off in the funds of hospitals which we suppose will be cited as a case in point." [The point is that the Darkest England Boom has taken large sums of money from the coffers of the older charities.] "The London hospitals spend every year six times the capital sum which the General asked for; the receipts this year show a falling off on last year of some £66,000. But if this is the year of 'Darkest England,' it is also the year in which 'More Hospital Scandals' has become a familiar heading in the Press, and which has shown us a blind and fatal obscurantism on the part of hospital officials, of which Mr. Burdett or his paper, has been the consistent champion and palliator." It is very easy to insert a paragraph headed "More Hospital Scandals," but it is not easy to justify the heading on the facts owing to the excellent management of our great voluntary hospitals in the present day. On the same evening the ollowing letter was addressed to the editor of the *Pall Mall Gazette*, who has not inserted it as we go to press this Tuesday night. We cannot, however, believe he will not possess the candour to print it, so prefer to conclude he is searching the records and will then speak. Hence we refrain from further comment this week:—

A Blind and Fatal Obscurantism.

*To the Editor of the "Pall Mall Gazette."*

The Lodge, Porchester Square, W.,
December 18th, 1891.

Sir,—I am sure I may claim the *Pall Mall Gazette* as an enlightened upholder of the principles of fair play and preciseness of statement. No thinking man or woman can fail to be impressed with a deep sense of the importance of providing adequately for the sick in hospitals in a great city like London. This sense impelled me to address you in the hope that you would aid in an endeavour to secure necessary funds in larger measure] to enable our voluntary hospitals to accomplish all the work cast upon them by the urgent needs of the suffering poor. You reply in a leading article in tonight's *Pall Mall* in which you state: (1) "This is the year of 'Darkest England' . . . in which 'More Hospital Scandals' has become a familiar heading in the press." (2) This year "has shown us a blind and fatal obscurantism on the part of hospital officials of which Mr. Burdett or his paper has been the consistent champion and palliator."

May I request you to be good enough to state:

(1) The instances or even the solitary instance during the year 1891 in which the heading "More Hospital Scandals" has been justified in the case of any voluntary hospital in London, or has even appeared in any newspaper of repute, except, perhaps, in the *Pall Mall Gazette*?

(2) Will you be good enough to quote the instance or instances "of blind and fatal obscurantism on the part of hospital officials" during the same year?

(3) Will you quote or refer me to a single article in which Mr. Burdett or his paper has championed or palliated the "blind and fatal obscurantism" aforesaid?—I am, Sir, your obedient servant, Henry C. Burdett.

